# Could Metabolic Syndrome, Lipodystrophy, and Aging Be Mesenchymal Stem Cell Exhaustion Syndromes?

**DOI:** 10.4061/2011/943216

**Published:** 2011-06-13

**Authors:** Eduardo Mansilla, Vanina Díaz Aquino, Daniel Zambón, Gustavo Horacio Marin, Karina Mártire, Gustavo Roque, Thomas Ichim, Neil H. Riordan, Amit Patel, Flavio Sturla, Gustavo Larsen, Rubén Spretz, Luis Núñez, Carlos Soratti, Ricardo Ibar, Michiel van Leeuwen, José María Tau, Hugo Drago, Alberto Maceira

**Affiliations:** ^1^Tissue Engineering, Regenerative Medicine and Cell Therapies Laboratory, CUCAIBA, Ministry of Health, Province of Buenos Aires, 1900 La Plata, Argentina; ^2^Lipid Clinic, Division of Endocrinology, Hospital Clinic, 08036 Barcelona, Spain; ^3^Division of Endometrial Regenerative Stem Cells, Medistem Inc., San Diego, CA 92122, USA; ^4^Division of Cardiothoracic Surgery, University of Utah, Salt Lake City, UT 84132, USA; ^5^Division Skin Bank, Burns Hospital, Buenos Aires City, C1424BSD, Argentina; ^6^LNK Chemsolutions, Division of Nanotechnology, Lincoln, NE 68521, USA; ^7^Bio-Target, Division of Nanotechnology, NE 68339, USA; ^8^The University of Chicago, Division of Nanotechnology, Chicago, IL 60637, USA; ^9^Nanovogue Inc., Division of Intelligent Matrices, Chicago, IL 60126-2731, USA; ^10^INCUCAI, Presidency, Ministry of Health, 2250-C1428BAJ Buenos Aires, Argentina; ^11^Vrije Universiteit, Burns Division, 1081 HV Amsterdam, The Netherlands

## Abstract

One of the most important and complex diseases of modern society is metabolic syndrome. This syndrome has not been completely understood, and therefore an effective treatment is not available yet. We propose a possible stem cell mechanism involved in the development of metabolic syndrome. This way of thinking lets us consider also other significant pathologies that could have similar etiopathogenic pathways, like lipodystrophic syndromes, progeria, and aging. All these clinical situations could be the consequence of a progressive and persistent stem cell exhaustion syndrome (SCES). The main outcome of this SCES would be an irreversible loss of the effective regenerative mesenchymal stem cells (MSCs) pools. In this way, the normal repairing capacities of the organism could become inefficient. Our point of view could open the possibility for a new strategy of treatment in metabolic syndrome, lipodystrophic syndromes, progeria, and even aging: stem cell therapies.

## 1. Introduction

Metabolic syndrome is recognized today as one of the most important causes of morbidity and mortality in the modern world [1, 2]. Metabolic syndrome is characterized by a variety of symptoms such as obesity with abundant visceral fat, dyslipidemia, carbon hydrates intolerance, insulin resistance and eventually type 2 diabetes, development of arterial hypertension, fat liver disease, sleep apnea, and atherosclerosis with high incidence of myocardial infarction and stroke [3–5]. Although many and different preventive and pharmacological strategies have been applied during the last two decades, the mortality rate of metabolic syndrome continues to be unacceptably high [6, 7]. Then, the central point to consider would be that its critical physiopathogenic pathway has not been discovered yet. As a consequence, it has not been possible so far to design the most appropriate and definitive treatment for it. In this context, it is essential to generate a new framework that could explain the main mechanism of this syndrome development and persistence, allowing then to an effective and enduring cure.

## 2. The Cellular Perspective

**Figure 1:**
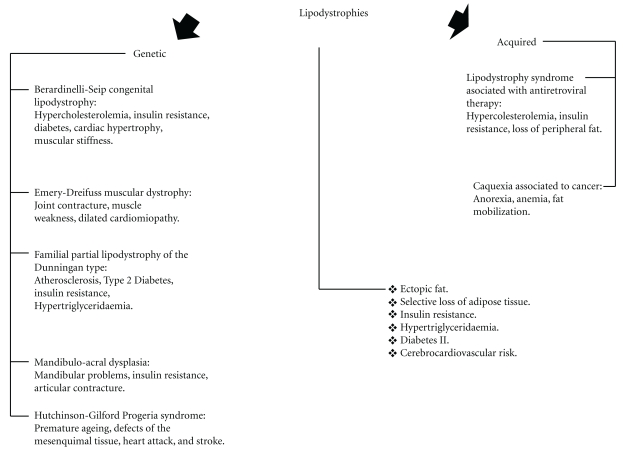
Lipodistrophic syndromes.

We propose to consider all these issues from a cellular perspective, which could open a pioneering vision for the interpretation and treatment of complex clinical situations such as metabolic syndrome, between many others. It is not generally known that metabolic syndrome is linked to lipodystrophies as much as to obesity [8, 9]. Congenital lipodystrophies (Berardinelli Seip syndrome, Emery-Dreifuss muscular dystrophy, and Dunnigan-type familial partial lipodystrophy) and acquired lipodystrophies (HIV-associated lipodystrophy, cachexia associated with neoplasias, among others) are characterized indeed by the loss of adipose tissue and also by insulin resistance, fat liver disease, dyslipidemia with hypertriglyceridemia, and many other manifestations of the metabolic syndrome [10–12] (Figure 1). In lipodystrophies, there is a continuous and severe loss of adipocytes by apoptosis leading to an inadequate metabolism of free fatty acids, generating severe organic consequences like lipotoxicity, which are closely related to development of metabolic syndrome [13, 14]. On the other hand, in obesity, there is also cellular damage but mainly produced by lipotoxicity, directly related to an excessive ingestion of calories and fats from the diet and by an overwhelmed system incapable of properly metabolizing them [15]. In this situation, hypertrophy and/or hyperproliferation of adipocytes would be the only physiological alleviating mechanism only for a short period of time [16]. Metabolic syndrome, lipodystrophies, and even progeria and aging could be more accurately explained by cellular mechanisms rather than by molecular and biochemical ones.

## 3. The Emergence of Adipocytes and the Perpetuation of Fat

The adipose tissue comprises one of the largest organs in the body. Even lean adult men and women have at least 3.0–4.5 kg of adipose tissue, and in individuals with severe obesity, adipose tissue can constitute 45 kg or more of body weight. The adipose organ is complex, with multiple depots of white adipose tissue involved in energy storage, hormone (adipokine) production, and local tissue architecture, as well as small depots of brown adipose tissue, required for energy expenditure to create heat (nonshivering thermogenesis) [17]. The potential to acquire new fat cells appears to be a permanent phenomenon in both animals and humans, before or after birth [18]. Therefore, proliferative adipocyte precursor cells must stand as ready to respond to increased demand for energy storage [19]. How adipocytes (fat cells) develop and where their progenitors come from, and for how long and under which circumstances they can provide sufficient support for more fat to be formed while maybe participating in other body functions, is a fundamental biological question with important ramifications for human health and disease. An increase in fat mass associated with obesity could only result from recruitment and differentiation of adipocyte progenitor cells. Despite the recognition of distinct progenitor populations in adipose tissue, it has been assumed that all white adipocytes and their progenitors arise solely from cells of mesenchymal origin [20]. Accumulating evidence suggests that adipocyte progenitors could proceed from bone marrow cells of mesenchymal lineage [21, 22]. Visceral adipose tissue associated with Metabolic Syndrome is a chemotactic niche, whereby mesenchymal stem cells can home to and differentiate into adipocytes to perpetuate its tissue formation [20]. The intertwined epidemics of obesity and diabetes demands an improved understanding of adipocytes and its progenitor cell biology. Adipose tissue mass can expand throughout adult life. Mesenchymal stem cells with a multilineage potential have been isolated from human adipose tissue. Their adipocyte differentiation has been thoroughly studied, and differentiated cells exhibit the unique feature of human adipocytes [22]. One paradigm supports the notion that adipocytes arise from mesenchymal stem cells (MSCs) by a sequential pathway of differentiation. When triggered by appropriate developmental cues, MSCs become committed to the adipocyte lineage. A better knowledge of MSC's differentiation pathways will surely allow the design of new therapeutic strategies for reconstruction of damaged tissues and for the control or prevention of risks associated with obesity in humans [17, 23, 24]. This process can be divided into two related steps: (1) determination, when multipotent mesenchymal stem cells commit to preadipocytes (these cells exhibit similar morphology compared to stem cells, but they are committed to the adipogenic lineage and are no longer able to transform into osteoblasts, myocytes, or chondrocytes and (2) differentiation, when preadipocytes become mature fat cells. This mechanism is tightly regulated at a molecular level by several transcription factors. Several members of the MAPKinases, bone morphogenic proteins, wingless-type MMTV integration site (Wnt) proteins, hedgehogs, delta/jagged proteins, fibroblastic growth factors, insulin, insulin-like growth factors, and transcriptional regulators of adipocyte and osteoblast differentiation including peroxisome proliferator-activated receptor-gamma and runt-related transcription factor 2 (Runx2) families have been shown to modify the steps of adipogenesis [23]. Despite the well-documented differences in the metabolic and biochemical properties among anatomically distinct depots of fat, the visceral fat contains adult mesenchymal stem cells with developmental potential similar to those isolated from subcutaneous fat in humans [21]. Thus, adipose precursors cells consist of fibroblast mesenchymal like multipotential stem cells generally termed adipose-derived stem cells (ASCs) and exhibit preadipocyte characteristics. They can be isolated, propagated in vitro, and induced to differentiate into adipocytes [25–27]. The adipose vasculature appears to function as a progenitor niche and may provide signals for adipocyte development. Stromal-vascular cells of adipose tissue are adipose precursor and its differentiation in vitro correspond to the sequence: adipoblast (unipotential cells), commitment preadipose cell (preadipocyte), terminal differentiation immature adipose cell, and terminal differentiation mature adipose cell (adipocyte) [28]. Also bone marrow progenitor- (BMP-) derived adipohematopoietic cells via the myeloid lineage have been mentioned as the adipocyte progenitors cells. In any way, these BMP-derived adipocytes could accumulate with age and occur in higher numbers in visceral than in subcutaneous fat, and in female versus male mice. BMP-derived adipocytes may, therefore, account in part for adipose depot heterogeneity and detrimental changes in adipose metabolism and inflammation with aging and adiposity [29]. The development of obesity not only depends on the balance between food intake and caloric utilization but also on the balance between white adipose tissue (WAT), which is the primary site of energy storage, and brown adipose tissue (BAT), which is specialized for energy expenditure. Considerable evidence now supports the view that BAT and WAT are distinct organs. In addition, some sites of white fat storage in the body are more closely linked than others to the metabolic complications of obesity, such as diabetes. White areas contain a variable amount of brown adipocytes, and their number varies with age, strain, and environmental conditions. Recent data have stressed the plasticity of the adipose organ in adult animals. Indeed, under peculiar conditions fully differentiated, white adipocytes can transdifferentiate into brown adipocytes, and vice versa. The ability of the adipose organ to interconvert its main cytotypes in order to meet changing metabolic needs is highly pertinent to the physiopathology of obesity and related to therapeutic strategies. The differentiation between white adipocyte and brown adipocyte lineages occurs in the earliest steps of the fetal development, and both phenotypes are acquired independently [30–33]. Fetal mesenchymal stem cells (fMSCs) can differentiate into brown and white adipocytes. The expression of key adipocyte regulators and markers during differentiation is similar to that in other human and murine adipocyte models, including induction of PPAR*γ*2 and FABP4. The preadipocyte marker, Pref-1, is induced early in differentiation and then declines markedly as the process continues, suggesting that fMSCs first acquire preadipocyte characteristics as they commit to the adipogenic lineage, prior to their differentiation into mature adipocytes. After adipogenic induction, some stem cell isolates differentiated into cells resembling brown adipocytes and others into white adipocytes. Importantly, these cells exhibited elevated basal UCP-1 expression. Thus, fMSCs represent a useful in vitro model for human adipogenesis and provide opportunities to study the stages prior to commitment to the adipocyte lineage. They also offer invaluable insights into the characteristics of human brown fat [34].

## 4. The Stem Cell Exhaustion Syndrome

**Figure 2:**
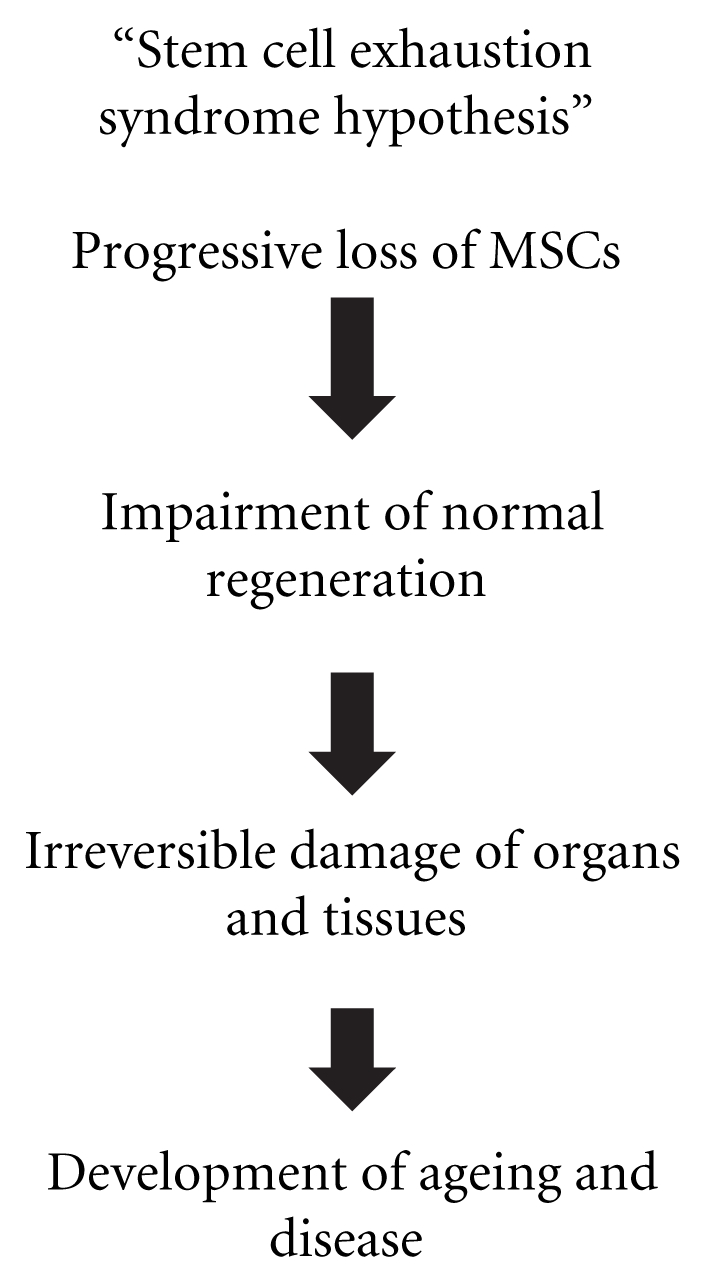
Stem cell exhaustion syndrome hypothesis.

In order to self-repair, living organisms have stem cells in central and peripheral locations which can be attracted to sites of injured tissues by “alarm signals” [35]. In this way, these cells proliferate, migrate, and accumulate in those damaged sites [36]. If this situation of “alarm” perpetuates, stem cells could be permanently exhausted from their original locations leading to irreversible disease (Figure 2). Basically, it could be a matter of stem cell quantity and effective availability mainly related to production and consumption in a certain time point when active regeneration is needed. The expected consequences of this situation could be the lack of an appropriate number of stem cells for further tissue replacement and regeneration and eventually the development of disease and aging. It is not completely clear yet if there could be a possible established, coordinated network or a dynamic connection as well as a biological equilibrium between all of these locations. This could finally lead to a constant traffic and exchange of stem cells among all of them in order to provide a perfect mechanism of stem cell provision and replenishment for normal repairment and the perpetuation of complex living organisms on Earth. Although there is not a definitive evidence for a possible alteration of this dynamic, involving an abnormal stem cell depletion kinetic mechanism, it could be interesting to hypothesize about these cell pathways that could open a new era of understanding of disease and therapeutics. For example, we could think that any alteration of this stem cell homeostasis by constant and repetitive trauma, physical hyperactivity, and chronic disease could provoke a persistent disequilibrium inside all these reserve locations. This could promote an irreversible and premature stem cell exhaustion syndrome (SCES), being impossible then for the organism to self-repair and survive.

## 5. MSCs: The Exhausted Stem Cell?

**Figure 3:**
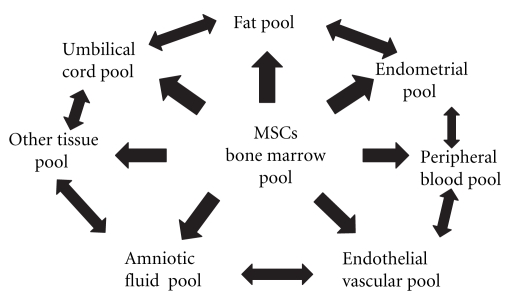
Mesenchymal stem cells pools as a coordinated network.

**Figure 4:**
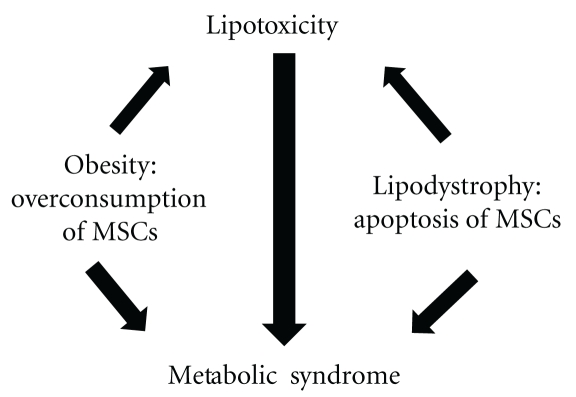
Metabolic syndrome in obesity and lipodistrophy.

Tissue and organ damage is constantly taking place in living organisms as a consequence of life itself, diseases, and trauma [37]. A decrease in the endogenous pools of progenitor cells, such as CD34 stem cells and endothelial progenitor cells (EPCs), has been demonstrated to contribute and accelerate the course of cardiovascular disease seen in metabolic syndrome. Several experimental studies have indicated a relevant contribution of these progenitor cells in reendothelization at sites of endothelial injury and in neovascularization at sites of ischaemia. The extent of the EPC pool negatively correlates with cumulative indexes of cardiovascular event risk, such as the Framingham risk score, and multiple risk factors act synergically in reducing EPC, increasing the risk for cardiovascular disease [38–40]. Mesenchymal stem cells (MSCs) are probably the most important specialized repairing cells [41, 42]. MSCs are adult stem cells with the capacity and potential of differentiation towards multiple tissue lineages such as adipose, bone, muscle, cartilage, skin, nervous system, and endothelium between many others [43, 44]. They can produce a large variety of growth factors, and they have immunomodulatory properties that allow them to avoid the immune rejection response when transplanted intra- and even interspecies [45, 46]. Although they reside mainly in the bone marrow (BM) and share with hematopoietic stem cells a similar microenvironment, they are phenotypically very different to them [47, 48]. Also, during the last few years, it has been possible to isolate them from many other sites, like dental pulp, endometrium, peripheral blood, umbilical cord, adipose tissue, and even amniotic fluid [49, 50]. Recent studies have also obtained MSCs from vascular vessels, being proposed that they could be found in the perivascular space throughout the whole body [51]. We will refer to all these precise anatomical locations where MSCs are stored as “MSCs pools,” being the bone marrow the central MSCs pool and the others the peripheral ones (Figure 3). In these pools, MSCs usually stay in a quiescent and undifferentiated state until they are called to proliferate and mobilize by “alarm signals” such as proinflammatory cytokines like INF-*α* and IL-6 among others and many growth factors like GM-CSF [52–54]. Then, it is possible to think that because of the supercaloric food intake of obese patients with metabolic syndrome, a high degree of proinflammatory substances could be produced and released in different microenvironments, specially the abdominal visceral fat one [55]. This could only lead to the perpetuation of this inflammatory state with a constant emission of “alarm signals,” proliferation, mobilization, and finally an endless sequestration of MSCs into the visceral fat depot [56]. Recently, a research group has found evidence of this adipotaxis phenomenon in an animal model, where the MSCs of the BM migrated attracted to the fat depot by TNF-Alfa [57]. This mechanism could give support to the idea of an abnormal migration of MSCs, in patients with metabolic syndrome, leading at some point to the mentioned irreversible impairment of tissue repairment (Figure 4).

## 6. MSCs Exhaustion and Aging

**Figure 5:**
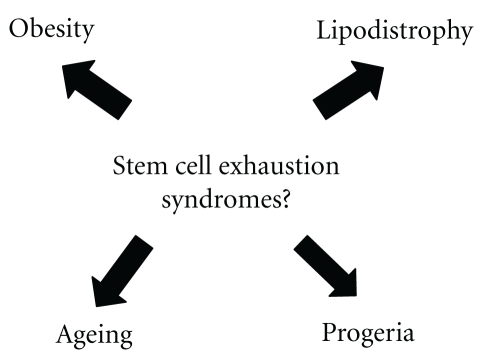
Pathologies propably caused by a stem cell exhaustion syndrome.

Metabolic syndrome incidence increases with the advancement of age [58, 59]. Human aging is another example of organ and tissue deterioration that could have a stem cell deficiency, very similar to that observed in metabolic syndrome. The classical human model of premature aging is the Hutchinson-Gilford Progeria syndrome (HGPS) [60, 61]. Progeria manifestations start at 18 months of age approximately, with alopecia, skeletal defects, distinctive facial appearance, and lipodystrophy [62, 63]. These patients also develop dyslipidemia and arterial hypertension [64]. Almost all of them have atherosclerosis as well as cardio and cerebrovascular disease by 13 years of age with premature death [65]. Progeria is produced by a mutation in the gene that codes for the protein of nuclear membrane Lamin A [66]. This mutation makes MSCs sensitive to apoptosis [67]. This issue could explain why many tissues of mesenchymal origin are specially affected in these patients [68]. HGPS is a pathology of segmental nature, in which the different tissues and organs exposed to a variety of different conditions such as mechanical stress are affected differently [69]. Tissues with different turnover rates would require a different number of stem cells for replacement. For example, hair and muscle cells should need to be replenished by MSCs more frequently than central nervous system ones [70]. In patients with progeria, stem cells are at least in principle irreversible damaged, suffering from early apoptosis [71]. Young peripheral tissues, especially those in continuous turnover, are probably more restricted of new replacing stem cells. In this way, progeria patients usually suffer as it was said from alopecia, vascular damage, and premature death by myocardial infarction or stroke [72, 73]. On the other hand, tissues with a slower turnover rate, such as central nervous system, suffer less notorious and more prolonged deterioration. Their pathology is not seen at all in progeria patients as they do not live long enough to be able to evidence damage of these tissues [74, 75]. An excessive cell turnover without the possibility of a concomitant cell replenishment mechanism, could lead to a slow but progressive deterioration usually seen in living organisms and known as “normal ageing” [76]. From this perspective, all these phenomena could be very similar to those observed in metabolic syndrome, lipodystrophic syndromes, and progeria. There is evidence that TNF-alfa progressively increases with age in adipose tissue which also rearranges itself and becomes dysfunctional with an inadequate response to insulin and increased production of cytokines [77]. This de novo proinflammatory generated environment is followed by a highly sensitive state of adipocytes to lipotoxicity [78] and a possible sequestration of a large number of MSCs especially from the BM central pool, which at the same time becomes progressively exhausted with the passing of time. “Normal aged” BM can be seen infiltrated by fat depots with a very reduced number of MSCs, having the significance of this phenomenon still unknown to date [79] (Figure 5). In other words, all these clinical situations could be explained by a stem cell exhaustion syndrome (SCES) causing an impaired regenerative potential. 

## 7. The New Paradigm: Cell Therapy

Stem cell restoration has already demonstrated therapeutic activities in certain systems. For example, it is known that after a stroke, endogenous stem cells are mobilized from the bone marrow in an attempt to heal the damaged neural tissue. Most interestingly, a recent study demonstrated that stroke patients who exhibit a high level of stem cell mobilization have better functional outcomes as opposed to patients with a lower mobilization [80]. Restoration of stem cell function has been studied in aging, in which senescent endothelium can be replaced by the addition of young endothelial progenitor cells. In animal models, this has been shown to “reverse” endothelial aging [81]. In patients administered GM-CSF in order to mobilize autologous bone marrow stem cells, improvements in endothelial function, as demonstrated by increased responsiveness of flow, have also been proven [82]. More “natural” means of mobilizing stem cells into the periphery include the use of food supplements. It has been reported that administration of “*StemEnhance*,” a commercially available food supplement made from cyanobacterium Aphanizomenon flos-aquae, induces a transient 18% increase in circulating CD34 cells over the period of one hour after consumption [83]. Another commercially available food supplement, “*Stem-Kine*,” has been demonstrated to induce a 50–100% increase of CD34 and endothelial progenitor cells in circulation for the observation period of over 2 weeks [84]. Given that similar increases in circulating stem cells have been associated with “health-inducing” activities such as exercise [85] and smoking cessation [86], it may be rationale to examine therapeutic effects of these supplements using functional endpoints. One critical point to consider is whether mobilization would accelerate exhaustion of stem cells in the bone marrow compartment. Given expression of telomerase in the bone marrow hematopoietic stem cells and its ability to be modulated by nutritional [87] and antioxidant interventions [88], it appears that this problem may be at least theoretically addressed. If a stem cell depletion kinetic abnormality (MSCs exhaustion syndrome) is true, then a stem cell therapy approach could be feasible. For instance, ex vivo expansion and reinfusion of MSCs from the patient's own or from allogeneic donors, as evidence shows that MSCs are not immunogenic at all [44], have been already tested in many clinical trials for different pathologies [89–93]. In the best case scenario, MSCs therapy could retard the onset of irreversible lesions associated with metabolic syndrome or at least partially improve those already present in the organism. Also, the development of bioartificial implants such as in the way of a fat transplant (autologous, allogeneic, or even xenogeneic) could be envisioned [94–96]. This could be an innovative way to provide a new pool of MSCs to the patients [17, 97]; a permanent fat transplant such as the one proposed here could also be enriched with ex vivo expanded MSCs, or even those previously made differentiated into brown adipocytes, becoming in this way an immune privileged niche for the cotransplantation and implantation of different kind of allogeneic cells, tissues, and organs needed for the better functioning and regeneration of living organisms, without the danger of rejection or the need of prolonged administration of immunosuppressive drugs [44, 98–101]. Adipose tissue transplantation has primarily been used as a tool to study physiology and for human reconstructive surgery [102]. Transplantation of adipose tissue is, however, now being explored as a possible tool to promote the beneficial metabolic effects of subcutaneous white adipose tissue and brown adipose tissue, as well as adipose-derived stem cells [103]. Data suggest that the upregulation of brown adipose tissue activity can contribute to a lean and metabolically healthy phenotype in humans; these findings also suggest that the transplantation or stimulation of brown adipose tissue might be used as a therapeutic approach to increase energy expenditure and lower white adipose tissue mass and improve the overall metabolism, also is used as a potential induction of beneficial metabolic effects and treatment of diseases, such as obesity, lipodystrophy, or cardiovascular disease. As the amount of endogenous brown adipose tissue is very limited, identification and manipulation of critical regulators of brown adipose tissue differentiation have been used to engineer brown adipose tissue in order to induce beneficial effects [104, 105]. Ultimately, the clinical applicability of adipose tissue transplantation for the treatment of obesity and metabolic disorders will reside in the achievable level of safety, reliability and efficacy compared with other treatments [17]. In this way, cell therapy undoubtedly will be the most promising therapeutic strategy of this century not only for metabolic syndrome, but also probably for lipodystrophies, progeria, aging, and many other diseases [106–110]. Finally, beyond generating new pharmacological and natural healthy nutritional regimens, we should start thinking in the provocative frontiers of stem cell mechanisms that we must necessarily explore in order to decrease, in the next few years, the deleterious effects of the above-mentioned pathologies. If a “stem cell exhaustion syndrome” could be the cause of all these morbid states, we will surely be able to generate the best modalities to prevent and treat them. Also, may be defeating at last, the erroneous idea of irreversible aging.
